# A case of intraabdominal tuberculosis masquerading as inguinal hernia: a diagnostic challenge

**DOI:** 10.1093/jscr/rjae741

**Published:** 2024-11-26

**Authors:** Sanem Yildirim, Yavuz Yigit, Atika Jabeen, Eslam Hussein Mohamed, Baha Hamdi Alkahlout

**Affiliations:** Department of Emergency Medicine, Hamad Medical Corporation, Al Rayyan Road, P.O. Box 3050, Doha, Qatar; Department of Emergency Medicine, Hamad Medical Corporation, Al Rayyan Road, P.O. Box 3050, Doha, Qatar; Blizard Institute, The Faculty of Medicine and Dentistry, Queen Mary University, The Blizard Building, 4 Newark Street, London, E1 2AT, United Kingdom; Department of Emergency Medicine, Hamad Medical Corporation, Al Rayyan Road, P.O. Box 3050, Doha, Qatar; Department of Emergency Medicine, Hamad Medical Corporation, Al Rayyan Road, P.O. Box 3050, Doha, Qatar; Department of Emergency Medicine, Hamad Medical Corporation, Al Rayyan Road, P.O. Box 3050, Doha, Qatar

**Keywords:** tuberculosis, intraabdominal tuberculosis, hernia, inguinal hernia, inguinal mass, iliopsoas abscess

## Abstract

Inguinal masses are common presentations in clinical practice, often attributed to hernias. However, atypical features may lead to diagnostic difficulties and delayed intervention. We present a case of a 32-year-old Ethiopian woman with a prolonged history of a growing groin mass 2 months following childbirth, her diagnosis potentially challenged by her recent obstetric history. Despite previous evaluations suggesting inguinal hernia, her symptoms worsened, prompting an emergency department visit. Further investigation revealed an unexpected diagnosis of intraabdominal tuberculosis, manifesting as a large iliopsoas abscess. This case underscores the importance of considering uncommon etiologies in the differential diagnosis of inguinal masses, particularly in high-risk populations with comparable situations, to ensure timely diagnosis and intervention.

## Introduction

Inguinal masses present diagnostic challenges due to their varied etiologies [1]. While about 5–11% of tuberculosis cases worldwide present as abdominal tuberculosis, its atypical presentation as an inguinal hernia is unusual and presents diagnostic difficulties [2, 3]. We present a unique case highlighting the diagnostic dilemma encountered in differentiating intraabdominal tuberculosis from inguinal hernia in a female with recent obstetric history. This presented additional challenges in the early detection and diagnosis, thus holding educational and learning value.

## Case report

A previously healthy 32-year-old African woman presented to the emergency department with a 5-day history of worsening left-sided abdominal pain and swelling. This pain was associated with a progressively enlarging groin mass, which began 2 months postpartum following the normal vaginal delivery of her third child. Additionally, she experienced debilitating pain in her left thigh and lower back, significantly impacting her mobility and daily activities.

Her postpartum gynecological assessment, which included a thorough vaginal examination, revealed a first-degree uterine prolapse. However, a pelvic ultrasound was unremarkable, providing little insight into the underlying cause of her symptoms. Initially, she was counseled, prescribed pain medications, and advised to follow up closely. Despite this initial treatment, her symptoms steadily worsened, with intermittent abdominal pain that was aggravated by even the most basic movements and exercise, increased tenderness of the enlarging groin mass, and increasing difficulty with ambulation.

During her first emergency department visit, there was a high clinical suspicion of an inguinal hernia, leading to a prompt referral to general surgery to rule out incarceration. She was advised to avoid any strenuous physical activity or heavy lifting and was discharged in stable condition with a prescription for non-steroidal anti-inflammatory drugs.

However, her condition persisted, prompting a return to the emergency department, this time with both surgical and medical consultations. At this juncture, she reported the development of debilitating night sweats, significant unintentional weight loss of over 5 kg, and generalized weakness that significantly impacted her overall well-being. Despite these alarming symptoms, she did not experience any fever, rigors, chills, nausea, vomiting, constipation, diarrhea, chest pain, cough, or difficulty breathing.

Upon examination, the patient was alert, oriented, generally well, and afebrile. Physical examination revealed a firm, tender, and irreducible mass measuring 20 by 15 cm in the left inguinal region. The mass was non-fluctuant, non-mobile, and adherent to underlying structures, with warmth to touch and hyperpigmentation. No cough impulse was present, and midline tenderness was noted on palpation over the lower back. Examination of the abdomen and right inguinal region was unremarkable.

**Figure 1:**
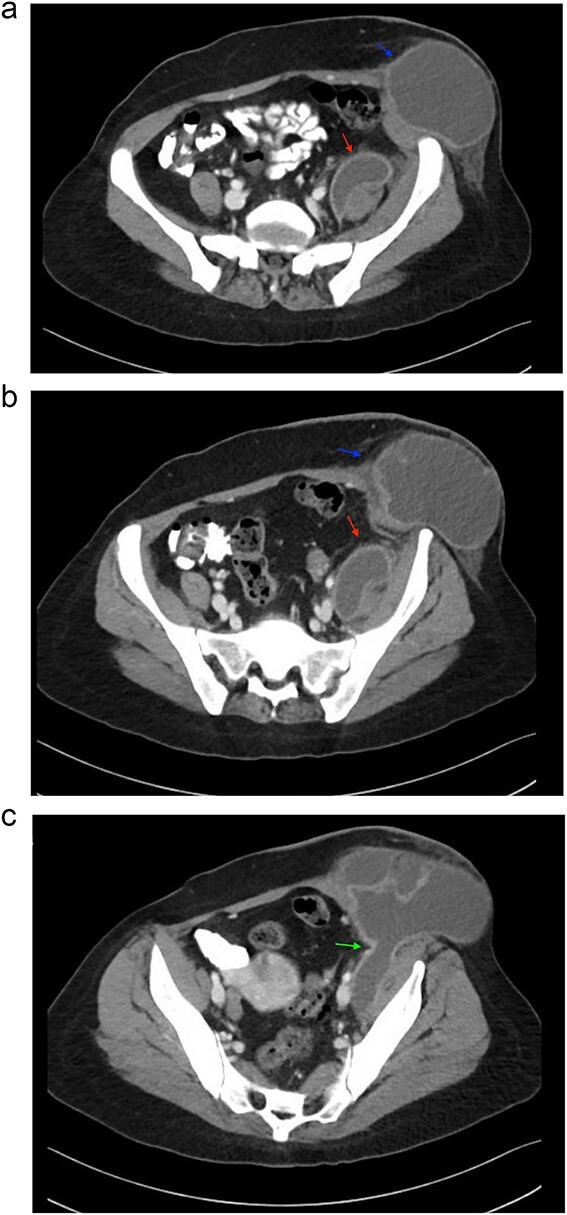
Sequential images of CT with contrast of the abdomen and pelvis demonstrating the pathology. (a) Slice 96—a small fluid collection in the left psoas muscle (lower arrow), and a low-density fluid collection in the left inguinal region (upper arrow), both with wall enhancement, consistent with abscess formation. (b) Slice 102—progression and enlargement of the iliopsoas (lower arrow) and inguinal (upper arrow) abscesses. (**c**) Slice 110—connection between the iliopsoas and inguinal abscesses, indicating advanced spread of infection (arrow).

The patient was admitted for further evaluation and management. Correlating her clinical history and physical examination findings with imaging studies, a CT scan of the abdomen revealed a 17 × 8 × 7 cm (transverse × craniocaudal × anteroposterior) multiloculated iliopsoas abscess tracking along the left inguinal canal, accompanied by destruction of the third and fourth lumbar vertebrae suggestive of a pyogenic etiology. Based on these findings, a diagnosis of Tuberculous spondylitis (Pott’s disease) with a secondary psoas abscess was made. Other nearby structures appeared unremarkable on imaging. Three sequential images of CT with contrast from the abdomen and pelvis series are provided, illustrating the pathology in detail (Fig. 1a–c).

Chest X-rays showed prominent broncho-vascular markings without focal consolidation, cardiomegaly, pleural effusion, or pneumothorax. A comprehensive tuberculosis workup was initiated. The purified protein derivative test was positive with an induration of 10 mm. Although the acid-fast bacilli (AFB) smear was negative, polymerase chain reaction testing was positive for *Mycobacterium tuberculosis* DNA. Subsequent AFB culture confirmed the presence of *M. tuberculosis* complex, sensitive to first-line anti-tuberculosis drugs.

The patient was commenced on a multi-drug anti-tubercular therapy regimen and discharged with a referral to the TB clinic for live, video-assisted Directly Observed Therapy Short-course (DOTS). Over the next 3 months of regular follow-up, she showed clinical improvement, with resolution of symptoms and reduction in abscess size.

## Discussion

Abdominal tuberculosis presenting as an inguinal hernia is exceedingly rare [2–10]. Early identification of infections is crucial as missing it delays treatment, worsening the condition and raising the risk of death [11]. Our case is unique due to several factors that contributed to a delayed diagnosis of abdominal tuberculosis presenting as an inguinal mass. Unlike other cases, the patient’s recent childbirth and postpartum complications, such as first-degree uterine prolapse and presumed postpartum cramps, led to the misattribution of her symptoms. Additionally, the absence of typical tuberculosis risk factors, such as recent travel history to endemic areas and known contact with TB patients, compounded the diagnostic challenge. Non-specific symptoms like lower back and thigh pain, coupled with the physical demands of managing three children, further obscured the diagnosis. Initial evaluations with vaginal ultrasound failed to reveal the condition, delaying the use of advanced imaging. The eventual diagnosis required a multidisciplinary approach involving emergency medicine, surgery, and infectious disease specialists, highlighting the complexity and rarity of such presentations. These factors collectively distinguish our case from others in the literature.

## Conclusion

To our knowledge, this is the first case of abdominal tuberculosis masquerading as an inguinal hernia in a female with recent obstetric history. This case highlights that intra-abdominal tuberculosis presenting as an inguinal mass poses diagnostic challenges especially in perspective of a recent obstetric history. Therefore, tuberculosis should always be in the differential diagnosis especially in cases of vague signs and symptoms with an endemic predilection. It also emphasizes the importance of multidisciplinary management as tuberculosis can present in a myriad of ways [2, 4]. A pre-operative suspicion of tuberculosis in cases of abdominal hernias is important to avoid unnecessary surgical procedures and improve outcomes in similar presentations.
